# Shapley-Additive-Explanations-Based Factor Analysis for Dengue Severity Prediction using Machine Learning

**DOI:** 10.3390/jimaging8090229

**Published:** 2022-08-26

**Authors:** Shihab Uddin Chowdhury, Sanjana Sayeed, Iktisad Rashid, Md. Golam Rabiul Alam, Abdul Kadar Muhammad Masum, M. Ali Akber Dewan

**Affiliations:** 1 Department of Computer Science and Engineering, Brac University, 66 Mohakhali, Dhaka 1212, Bangladesh; 2 Department of Computer Science and Engineering, International Islamic University Chittagong, Chittagong 4318, Bangladesh; 3 School of Computing and Information Systems, Athabasca University, 1 University Dr, Athabasca, AB T9S 3A3, Canada

**Keywords:** dengue, Dengue Shock Syndrome, Dengue Haemorrhagic Fever, Shapley Additive Explanation, supervised, unsupervised, hierarchical clustering, XGBoosting, clinical data

## Abstract

Dengue is a viral disease that primarily affects tropical and subtropical regions and is especially prevalent in South-East Asia. This mosquito-borne disease sometimes triggers nationwide epidemics, which results in a large number of fatalities. The development of Dengue Haemorrhagic Fever (DHF) is where most cases occur, and a large portion of them are detected among children under the age of ten, with severe conditions often progressing to a critical state known as Dengue Shock Syndrome (DSS). In this study, we analysed two separate datasets from two different countries– Vietnam and Bangladesh, which we referred as VDengu and BDengue, respectively. For the VDengu dataset, as it was structured, supervised learning models were effective for predictive analysis, among which, the decision tree classifier XGBoost in particular produced the best outcome. Furthermore, Shapley Additive Explanation (SHAP) was used over the XGBoost model to assess the significance of individual attributes of the dataset. Among the significant attributes, we applied the SHAP dependence plot to identify the range for each attribute against the number of DHF or DSS cases. In parallel, the dataset from Bangladesh was unstructured; therefore, we applied an unsupervised learning technique, i.e., hierarchical clustering, to find clusters of vital blood components of the patients according to their complete blood count reports. The clusters were further analysed to find the attributes in the dataset that led to DSS or DHF.

## 1. Introduction

Over the last several years, the number of cases of dengue fever has been increased dramatically all around the world [1,2]. Dengue fever is an acute febrile viral disease carried by Aedes mosquitoes carrying one of the four dengue virus serotypes. According to a recent study, 390 million dengue illnesses occur each year, and dengue transmission is omnipresent across the tropics, with a high risk in America and Asia [3]. Dengue cases are prevalent throughout Southeast Asia, and its epidemic varies throughout the regions every year [4]. Most subtropical countries have made tremendous progress in the management of communicable diseases. However, these countries still have problems managing dengue cases, sometimes scaling to epidemic levels. A special problem is a vasculopathy marked by endothelial dysfunction and plasma leakage that occurs several days after the disease arose, often throughout the time of defervescence; this is much more severe in case of children and can often cause hypovolaemic shocks, which is known as Dengue Shock Syndrome (DSS) [5,6,7]. According to the World Health Organisation (WHO), typical Dengue Fever (DF) is defined by a platelet count of just under 150,000 cells/mm${}^{3}$ and an increasing hematocrit level of 5–10% with no plasma leakage and leukopenia, which is referred to as a WBC count and is often less than 5000 cells/mm${}^{3}$. In the event of DHF or more severe DSS, it is defined by thrombocytopenia of less than 100,000 cells/mm${}^{3}$ and hematocrit concentrations higher than 20% [8,9,10].

Our motivation in this research study is to see which blood components vary when a patient proceeds towards the dengue severity state. Given that we know the blood components that vary during this early stage, we can take precautions beforehand and healthcare professionals can take measures to provide appropriate treatment before the patient reaches to a critical state. We feel that improving collaborations on the severity analysis of dengue sickness by integrating clinical and basic researches is critical in tropical and subtropical countries, where the disease affects approximately half of the world’s population [11]. However, there has been a significant gap due to difficulties in collaboration, issues with data availability, limited financial resources, limited human resources, and historical context. Thus, the ultimate control of dengue could be done by an integrated, multidisciplinary and multinational research program to acknowledging the gap in dengue diagnosis.

To bridge this gap and address some of the issues that can help to reduce the misclassification of dengue severity, we analysed different attributes of blood components that leads a patient towards Dengue Haemorrhagic Fever (DHF) or a Dengue Shock Syndrome (DSS), especially in the subtropical and tropical regions. The contributions of our study are as follows:

- Several supervised learning approaches, such as random forest, decision tree, XGBoosting, and AdaBoosting, were applied to our dataset, with the XGBoost classifier model proving to be the best fitting algorithm with the highest accuracy for determining dengue severity [11,12,13]. Shapely Additive Explanations (SHAP) were then run on top of the XGBoost to quantify the contribution of each attribute in the dataset to dengue severity. SHAP is a game-theoretic technique for explaining the output of any machine learning model [14,15]. This method aided in the extraction of critical aspects that were mostly responsible for driving a patient to DHF or DSS. Next, the SHAP dependence plot was presented between the significant attributes, which suggested that patients having a platelets count of less than 100,000 (cells/mm${}^{3}$) and hematocrit levels greater than 20% have a higher chance of leading towards critical conditions. Thus, the early detection of the above-mentioned criteria will help to recognise the severity of dengue and increase the scope of giving proper treatment to the patient.
- The study with the datasets of Bangladesh (BDengue) and Vietnam (BDengue) showed a close association between different blood components regarding predicting severity among dengue patients. The BDengue dataset contains unstructured data. As a result, we considered implementing unsupervised learning, which is called agglomerative hierarchical clustering. After analysing the data, it was found that there exists a strong relationship of DSS or DHF with the patient’s platelet count and HCT concentration. Based on this study, a similar pattern is observed among dengue-infected patients across the subtropical regions.
- Among four of the serotypes, DENV-1 and DENV-2 were found to be significantly associated with an increased risk of DSS and DHF in the VDengue dataset. Therefore, it can be said that, apart from the aforementioned blood components, the serotype also plays a role in dengue severity. As a result, the early detection of serotypes could be an important approach in reducing the number of severe outcomes of dengue cases.

The remainder of this paper is organised as follows. Section 2 contains a description of the related works, and various methods and techniques that fall under the same domain. Section 3 consists of the working principles that we have used in our study and a description of both the VDengue and BDengue datasets, along with the data construction. Section 4 explains the techniques that we used to handle the missing values to bring consistency to our data. Section 4 contains the necessary descriptions regarding how we extracted the right features or properties from our raw data to analyse the dengue severity among dengue patients. Section 6 consists of the methods and algorithms that we have proposed that are the best fit for our datasets. Section 7 consists of the results that we obtained after analysing our datasets to predict dengue severity among patients. The entire research is ended and summarised in Section 8 by demonstrating the similarities between aspects of the two specified subtropical nations, as well as limitations and future opportunities.

## 2. Related Work

In [16], Sanjana Das and Abha Thakral used an R predictive analysis approach to foretell dengue and malaria disease. They conducted a time-series analysis of the data by using R with a generic X-Y charting and linear regression. The main goal in their time series analysis was to forecast the future values of the series. They also used a generic function for X-Y plotting in their data analysis, where the different lines in the plot reflected different years where the cases occurred during the period from 2010 to 2015.

In [5], the authors monitored and analyzed the platelet and haematocrit count in blood from children who had laboratory-confirmed dengue to predict DSS. They also took the data of Vietnamese children aged from 5–15 years admitted to the Hospital with clinically suspected dengue cases between 2001 and 2009. All the data in the dataset comprised laboratory-confirmed dengue cases within 1–4 days of illness. For both univariate and multivariate analyses, logistic regression was the dominant statistical model in this research study. The predictive values of daily haematocrit and platelet counts were tested using graphs and independent regression models fitted for each day of sickness.

In [17], the authors took a total of 515 patients’ data to predict the cause of DHF by observing peripheral values of the blood count. Data were evaluated utilizing IBM-SPSS version 16 of the statistical package. The student test results were used to investigate the variations between the mean peripheral blood variables in the acute stage and the critical phase. Linear regression was used to investigate trends in parameters over the duration of the epidemic.

In [18], the authors collected 530 dengue-infected patients’ data from Nawaloka Hospital Sri Lanka (NH) and studied their lymphocyte count to correlate with dengue severity infection. They used descriptive statistics to be derived and articulated as key pattern and frequency indicators. P-values obtained by means of the Student’s *t*-test were used to evaluate averages among two classes.

## 3. Methodology

### 3.1. Description of VDengue Dataset

In this study, we used the Vietnam’s dataset from [5] which we referred as VDengue. This dataset contains clinical data of 2301 children suffering from dengue in the Cohort. The patients were admitted in a hospital in Vietnam between the years 2001 and 2009 [5]. Among the 2301 patients, 143 (i.e., 6.21%) progressed to Dengue Shock Syndrome (DSS), and the remaining 2158 (i.e., 93.79%) did not reach to DSS. However, they suffered from normal dengue diseases. The patients age in this dataset ranged between 5 and 15 years old. The dataset contains information of the patients, such as age, gender, weight, temperature, and pulse rate on the day of their admission in the hospital. It was observed that most of the patients had confirmed dengue between 1 and 4 days of their admission. The dataset also contains information regarding the platelet counts (cell/mm${}^{3}$) and haematocrit concentration (%) on the day of their admission. The serology, serotype, and tourniquet test results were also added to the database. Beside this, some of the significant symptoms of dengue, such as abdominal pain, tiredness, vomit, and mucosal bleeding, were recorded in order to determine the severity of the patient. The minimum platelet count and maximum haemoconcentration between days 3 to 8 were recorded in the dataset.

**Figure 1:**
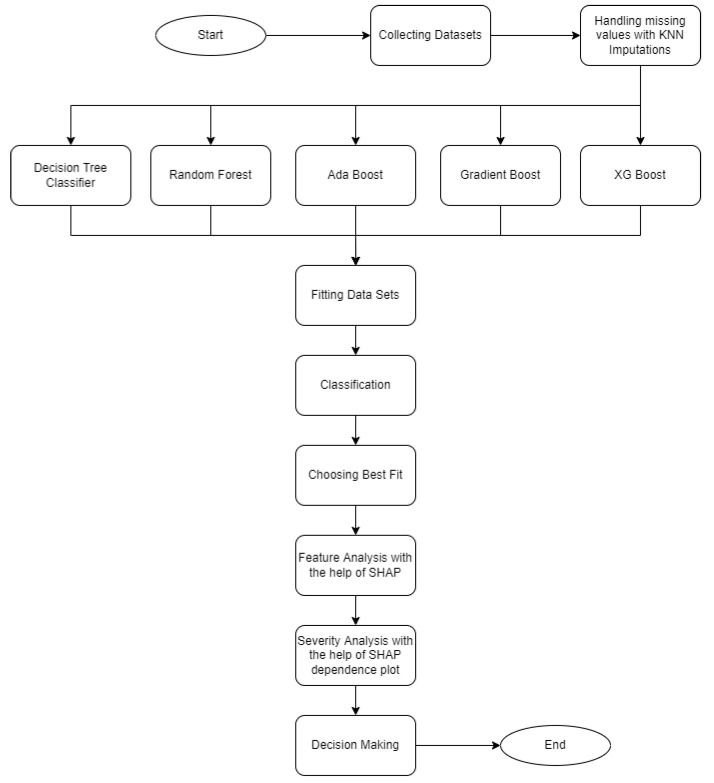
Top level overview of the dengue prediction model (VDengue dataset).

The workflow diagram for this dataset analysis is shown in Figure 1.

In this dataset, there were some missing values that we handled using KNN imputation method. The refined data were then passed through the following five models: decision tree classifier, random forest, AdaBoost, Gradient Boost, and XGBoost classifier. We recorded the sensitivity, specificity, misclassification, precision, f1_score, PPV, and NPV of the classification models, where the XGBoost gave the best performance. Next, the XGBoost classifier model was sent to the SHAP tree explainer to reveal the important features based on the predicted output, i.e., “shock”. Finally, the SHAP dependence plot was used among the important features to find which variable contributed more to determine the severity of dengue among the patients.

The different features for the VDengue dataset are shown in Table 1.

The target variable for the VDengue dataset is the “shock” column, which contains the binary value ’Yes/No’ for patients who either went into shock or didn’t. As the XGBoost classifier gave the highest accuracy, this model was sent to the SHAP tree explainer to identify the importance of features based on the predicted output, i.e., “shock”.

**Figure 2:**
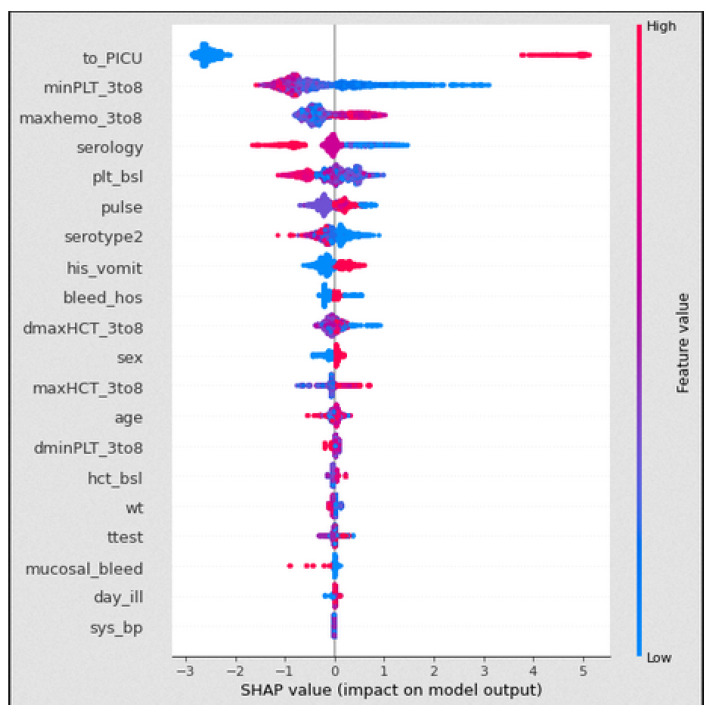
Summary plot of different features of the datasets using SHAP values on XGBoost classifier model.

The Figure 2 plot uses the SHAP values obtained from the XGBoost classifier model, which identifies the important features [15]. The horizontal axis contains the SHAP values of our predicted output, i.e., shock. The positive values along the right side of the horizontal axis refers to shock positive (1) and the negative values on the left side refers to shock negative (0). The vertical axis is determined by the features from our dataset, where the most important features are on the top and the least important features are at the bottom. The threshold colours red, blue, deep blue defines a high value, a medium value, and a low value, respectively.

- to_PICU: When to_PICU is high, the patients have reached the paediatric unit, then the shock syndrome is positive (1). When to_PICU is low, the shock syndrome is negative (0).
- minPLT_3to8: When the minimum platelet count of 3 to 8 days from enrolment is low (or blue value), the shock value is positive (1). When the minimum platelet count is mid to high (i.e., deep blue to red), the shock value is negative (0).
- maxhemo_3to8: When the maximum haemoconcentration value of 3 to 8 days from enrolment is high (or red), the shock is considered as positive (1). When the maximum haemoconcentration value is mid to low (deep blue or light blue color), the shock value is considered as negative (0).
- serology: When the serology is high, the shock is considered as negative (0). When it is low, the shock is considered as positive (1).
- plt_bsl: When the platelets count at the day of enrolment is high, the shock is considered as negative (0). When it is low, the shock is considered as positive (1).
- pulse: When the pulse rate of patients in our datasets are mid value, they did not reach shock, i.e., shock-negative (0), but a pulse rate with a low or high value is shock-positive (1).
- serotype_2: A high to mid value is shock-negative (0), and a low value is shock-positive (1).
- his_vomit: When the patients do not show any sign of vomit on the day of enrolment, then it appears that the patients are shock-negative (0), and if they show a tendency to vomit, they appear to be shock-positive (1).
- bleed_hos: When the patients at the day of enrolment do not show any symptoms of bleeding, they have a greater probability to not reach shock, but patients with bleeding symptoms appear to shock.
- maxHCT_3to8: When the maximum haematocrit count of patients 3 to 8 days from enrolment is high or red, then their shock value is positive (1), and when the maximum haematocrit value is mid to low or deep blue or light blue, then their shock value is negative (0).

### 3.2. Description of BDengue Dataset

We used another dataset collected from two hospitals (Dr. M. R. Khan Shishu Hospital and Central Hospital) in Bangladesh, which we referred as BDengue. In this dataset, the data collected from the Dr. M. R. Khan Shishu Hospital contains 69 patients who were aged between 8 months and 15 years. The dataset contains WBC, platelets count, lymphocytes, monocytes, etc., that a normal blood test report contains, and the symptoms that were visible at an early stage when the diseases were detected. This dataset also contains NSI, IgM, and IgG test results. The use of BDengue dataset was approved and authorised by the hospitals under condition of keeping the patients identity anonymous and confidential. The patients data are owned by the hospitals and are consented by the patients. We acknowledged this in our acknowledgment section.

**Figure 3:**
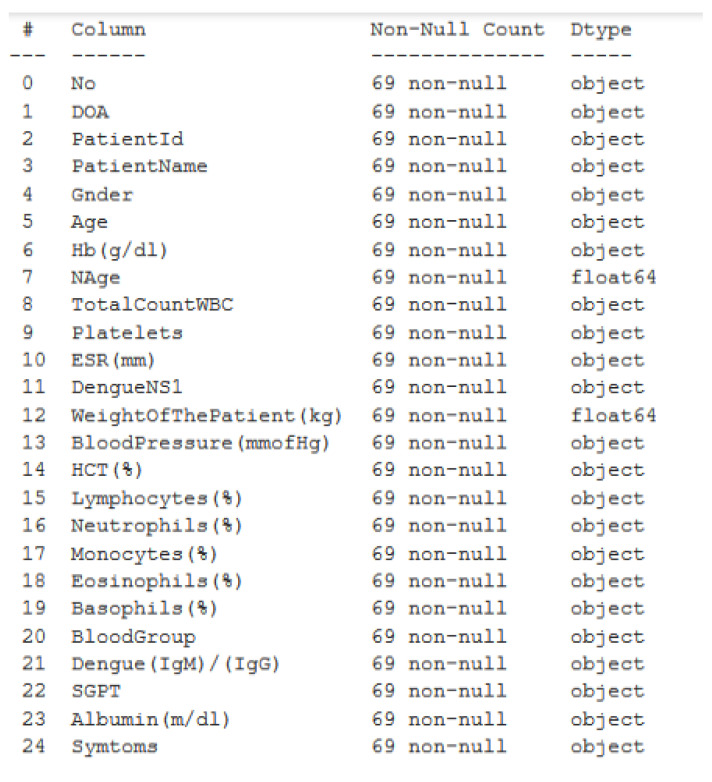
Showing attributes of the Shishu Hospital dataset.

Table 2 and Figure 3 show the attributes of the dataset that we gathered from the Dr. M. R. Khan Shishu Hospital, which are as follows:

Similarly the data collected from the Central Hospital contains a haematology report and laboratory report of a dengue test of around 100 patients of different ages. Table 3 shows the attributes of the BDengue dataset. In our study, we aggregated the dengue patient’s information from the above two hospitals and developed a model by analysing the phenotypic characteristics of the patient by merging the file in one dataset and selecting similar features among the datasets [19].

For the BDengue dataset, there was no target column because of the unstructured data. Therefore, we proceeded with unsupervised learning. We applied hierarchical clustering to form identical clusters and analysed the behaviour of those clusters.

**Figure 4:**
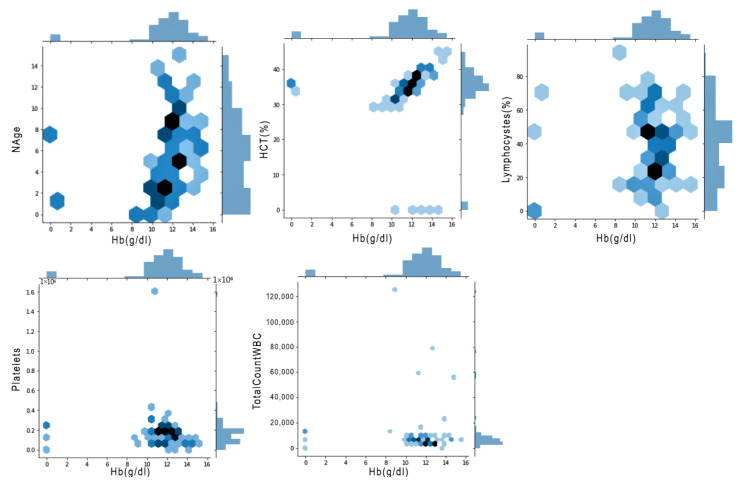
Bi-variant Relation between different features of the BDengue dataset.

Figure 4 shows a bi-variant analysis between haemoglobin and other features, such as platelets, HCT, WBC, and lymphocytes. The plot was used to calculate two events occurring at the same point in time. In Figure 4, five different plots show a correlation among different components of blood. Here, we are comparing platelets and HCT. WBC and lymphocytes are compared with respect to haemoglobin. This creates a regression line between two events and computes a probability. The darker blue region in the Figure refers to a higher concentration.

**Figure 5:**
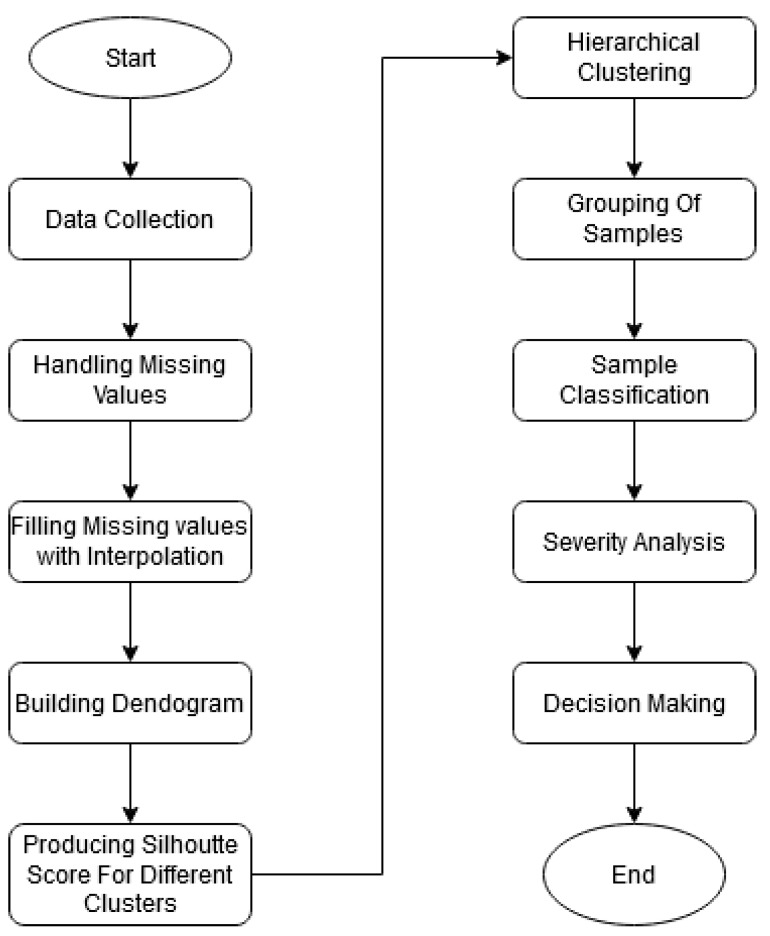
Top level overview of the dengue prediction model (BDengue dataset).

The working flow diagram for the BDengue dataset is shown in Figure 5.

In Figure 5, the working procedure on BDengue dataset is described. As we did not have enough data from a single source, we merged data from two hospitals with the common attributes in this dataset. The missing values were handled using an interpolation method in the dataset. As the BDengue dataset was not very structured and there was no output variable, we decided to apply unsupervised learning on the dataset. Agglomerative hierarchical clustering was conducted to form a cluster hierarchy, which was demonstrated in a dendrogram. After that, silhouette scores were determined to evaluate the quality of the clusters, and it was found that the BDengue dataset could be well divided into two clusters. The mean and standard deviation of the features for both clusters were determined. Finally, the two clusters were examined further to analyse whether or not patients from either cluster had progressed to severity.

## 4. Missing Value Imputation

### 4.1. KNN Imputation

The VDengue dataset has 2301 rows, but there were some missing values. Thus, the dataset required some preprocessing. We tested two imputation algorithms–multiple imputation by chained equations (MICE) algorithm and KNN imputer, where the KNN imputer worked well for our dataset. Thus it was finally used to handle the missing values. The KNN imputer model is basically a regressive model for predicting missing values. Input variables are required to be numerical [20]. However, in our dataset, among 24 columns, 11 of them contain categorical values. Thus, those specific categorical columns were converted to numerical values using a label encoder. When all the values of each column were converted into numbers, the KNN imputer was used to fill the missing values using the KNN algorithm. This imputation method works by searching the whole dataset to find similar instances in order to fill the missing data. The KNN identifies neighbouring points in the dataset by calculating the distance using the Euclidean formula [21]. The formula for the Euclidean distance is given below:

(1) $$d\left(x,y\right)=\sqrt{\sum_{i=1}^{n}{\left(y_{i}-x_{i}\right)}^{2}}$$

where

*x*, *y* = two points in Euclidean *n*-space;
*y*${}_{i}$, *x*${}_{i}$ = Euclidean vectors, starting from the origin of the space, i.e., the initial point;
*n* = *n*-space [22].

### 4.2. Interpolation

As the amount of data for the BDengue dataset was very small, we did not want to drop the rows with missing values. We rather used interpolation to fill the missing values. Interpolation is a mathematical analysis that adjusts a function to our dataset and, using that function, the missing value is deduced [22,23]. The interpolation formula that was used to fill the missing values in our BDengue dataset is as follows:

(2) $$y=y_{1}+\left(x-x_{1}\right)\frac{\left(y_{2}-y_{1}\right)}{\left(x_{2}-x_{1}\right)}$$

where

*y* = linear interpolation value;
*x* = independent variable;
*x*${}_{1}$, *y*${}_{1}$ = values of the function at one point;
*x*${}_{2}$, *y*${}_{2}$ = values of the function at another point [24].

## 5. Feature Engineering

**Figure 6:**
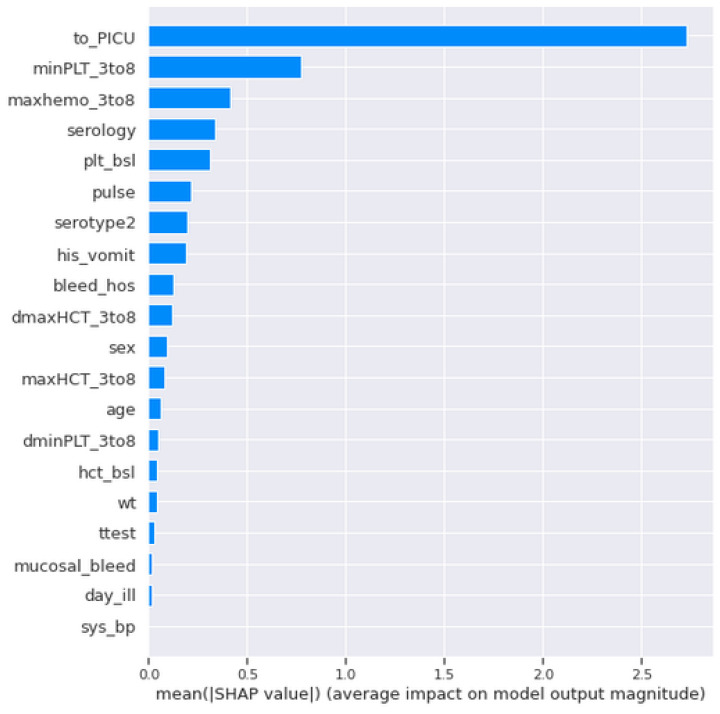
Bar diagram showing the importance of different features for the VDengue Dataset.

For the VDengue dataset, we used a summary plot. For all the features and samples in the selected range, the plot aggregates Shapley Additive Explanations (SHAP) values. SHAP values are sorted in a way where the most important feature is at the top of the list. The important features of the dataset are shown with the help of a bar diagram, where the features are categorised according to their precedence. The bar diagram with the important features for the VDengue dataset is shown in Figure 6.

For the BDengue dataset, we merged the data collected from two different hospitals into a single dataset consisting of 169 patients based on common attributes. The following are the features that we selected:

- Sex;
- Age (yr);
- Hb (g/dL);
- HCT (%);
- Platelets (cells/mm${}^{3}$);
- WBC (/cmm);
- Lymphocytes (%);
- Neutrophils (%);
- Monocytes (%).

All the 169 patients in the dataset were dengue positive. For instance, the unit for platelet in the Central Hospital was `K/L’, which we converted into `cells/mm${}^{3}$’ to make it similar to Dr. M. R. Khan Shishu Hospital. We also applied the ExtraTreeClassifier model to the newly created dataset to find the most relevant attributes among the features [25].

**Figure 7:**
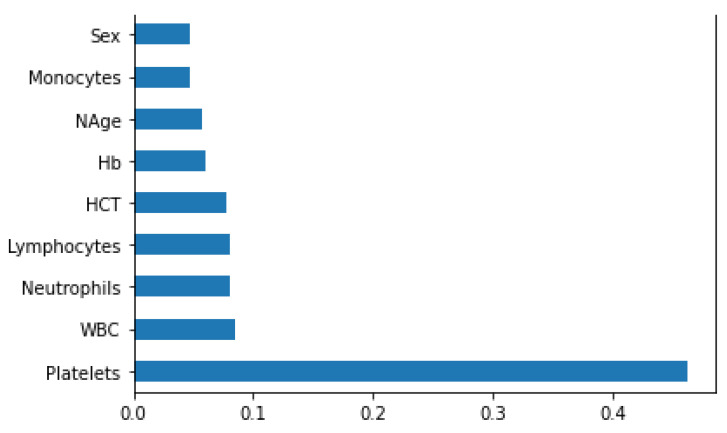
Bar diagram showing important features of the BDengue dataset.

In Figure 7, it is seen that the platelets have the highest score, so it is a more relevant or important feature to find the severity among the patients. Apart from the platelets, the HCT, lymphocytes, neutrophils, and WBC are also found to be important.

## 6. Proposed Model

As we mentioned earlier, we have used two datasets in this research study: VDengue and BDengue. Since the VDengue dataset was labeled and structured, we used supervised learning. On the other hand, since the BDengue dataset was unstructured, we used unsupervised learning.

### 6.1. Supervised Learning

In the VDengue dataset, the target column “shock” produces negative (no) and positive (yes) results. Since the output variable is categorical, several supervised classification methods were used to identify the category of the new observations. Furthermore, the dataset was nonlinear and featured categorical target variables, due to which, the decision tree model was implemented. As the depth of the tree increases, the accuracy improves as well. To further improve the performance of the model, we experimented with boosting algorithms, such as the XGBoost and AdaBoosting classifiers, where the XGBoost gave the highest accuracy as it has an inbuilt regularisation property that minimizes overfitting.

Since the classification models, such as decision tree and random forest, were fitted on our VDengue dataset, we applied both the criteria gini and entropy with maximum depth ranging from 1 to 20. The data were fitted for each depth with both criterion, and were tested to find whether the model can predict shock symptom accurately. Furthermore, boosting algorithms, such as AdaBoosting, XGBoosting, and Gradient Boosting, were used. To optimize the model the hyper-parameters, such as the learning rate was adjusted between the ranges of 0.05 and 0.75, at an interval of 0.025 and maximum depth ranging between 1 and 20. The colsample bytree, alpha, and n estimators of the aforementioned algorithms were also tuned for the training dataset. The log loss curve was finally drawn to determine if the model’s prediction in finding the severity among dengue-infected patients was correct. The classification report (Table 4 and Table 5) containing different metrics, such as sensitivity, specificity, misclassification, precision, f1_Score, PPV, and NPV, were further analysed to see which model fits the best with the VDengue dataset to predict the severity among the dengue-infected patients [26,27].

The formula for finding the accuracy, sensitivity, specificity, precision, recall, and f1_score are as follows:

- Accuracy: (True Positive + True Negative)/(True Positive + False Positive + True Negative + False negative);
- Sensitivity: True Positive/(True Positive + False Negative);
- Specificity: True Negative/(True Negative + False Positive);
- Precision: True Positive/(True Positive + False Positive);
- Recall: True Positive/(True Positive + False Negative);
- f1_score: 2 * ((Precision * Recall)/(Precision + Recall)).

The predictive algorithms shown in Table 5 were fitted on the VDengue dataset. The whole VDengue dataset was splitted into a 70% training and 30% testing set randomly and was fitted to the model. After fitting the dataset, random rows from the testing dataset were selected to see the predictive output. These outputs were validated with the original data to check the correctness of the model.

### 6.2. Unsupervised Learning

We used an unsupervised model for the BDengue dataset because this dataset has no output variable. As we implemented unsupervised learning, no training for the model was needed. The BDengue dataset was fed into the agglomerative hierarchical clustering model, which considered each observation as a separate cluster. This algorithm then iteratively finds the closer clusters and merged them into a single cluster. Finally, the clusters formed are further analysed to determine which cluster of patients progressed to severity.

## 7. Result and Analysis

### 7.1. Analysis on the VDengue Dataset

As we mentioned earlier, among the supervised learning we applied, the XGBoost classifier was found to be best performing on our dataset. It is also reflected in Table 4 and Table 5. For the XGBoost model, the hyper parameters were tuned, such as objective = binary logistic, colsample_bytree = 0.3, learning rate = 1, max_depth= 9, alpha = 10, and n_estimators = 10, to achieve a better performance.

**Figure 8:**
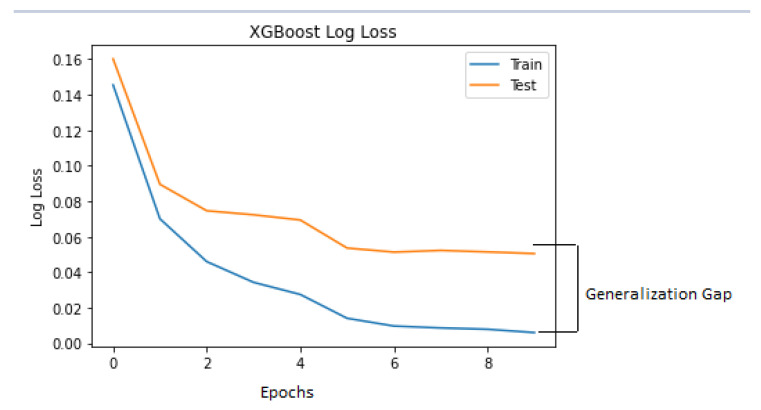
XGBoost log loss curve.

The size of the epochs was taken as equal to the length of the evaluation set. Figure 8 is the log loss which indicates the model behaviour on the train and test dataset over the training epochs. As the generalisation gap was small between the training and testing log-loss curve, it could be said that the XGBoost classifier model had a good fit on the VDengue dataset in predicting the dengue severity.

**Figure 9:**
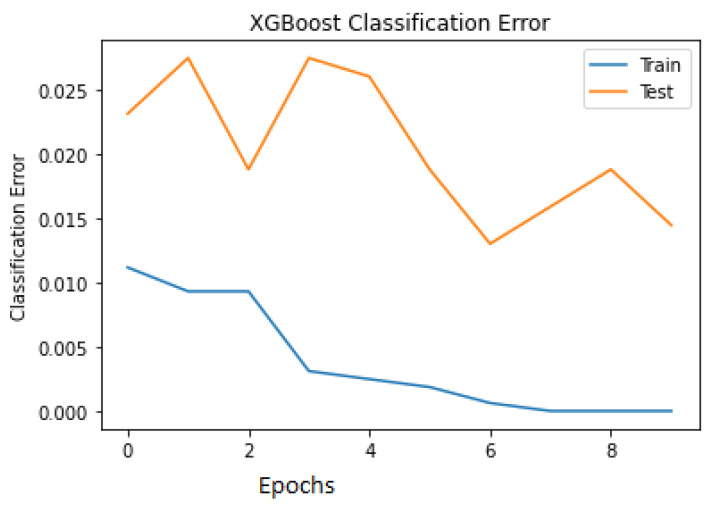
XGBoost classification error curve.

The classification errors for both the train and test are plotted in Figure 9 to visualise the misclassification among the data points. It is seen in the last epoch that the number of misclassified samples during training is close to 0, and during testing, it is close to 0.05.

**Figure 10:**
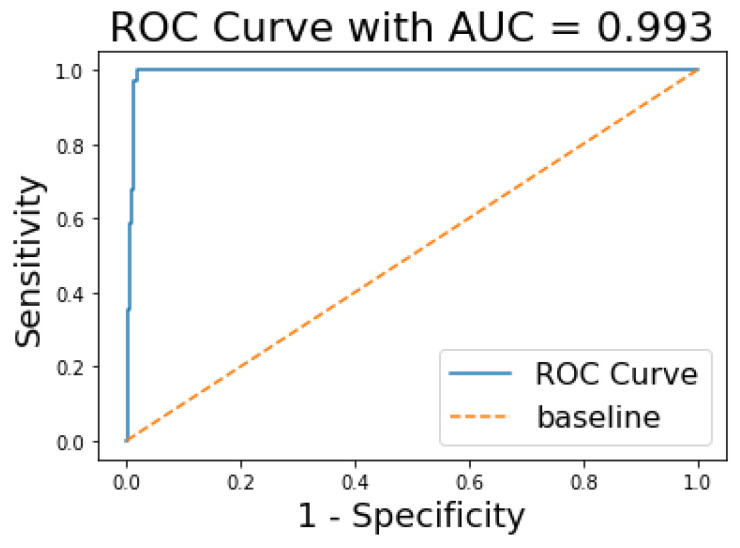
ROC and AUC Curve.

The ROC and AUC curves were further assessed to see the performance of our model. The AUC curve summarises the performance and gives a metric that lies between 0 and 1. The value tends to 1 for a high performing classifier and 0 for a low performing classifier. From Figure 10, we can see that the AUC for the XGBoost model is 0.993.

In the VDengue dataset, among all the features, the to_PICU, maxHCT_3to8, minPLT_3to8, maxhemo_3to8, and serotype2 are considered to be the most important features as described in Section 3, and the analysis was done by taking all these features under consideration. The influence of the aforementioned variables on the predictions given by the XGBoost classifier, which was the best fitted model for the VDengue dataset, was depicted using a SHAP dependency plot. This plot assisted us in analysing the factors that have a higher chance of causing shock, i.e., severity. A SHAP value greater than zero shows that the prediction result is positive, indicating that the patient has gone into shock, whereas a value less than zero suggests that the patient has not yet reached to shock level or acquired DSS [15].

- TO_PICU AND MAXHEMO_3TO8 AND MAXHCT_3TO8 AND HCT_BSL:

The term haemoconcentration refers to a drop in plasma volume that is accompanied by an increase in red blood cell concentration. With the increase in haemoconcentration, the blood viscosity is also increased and causes fever. The DHF and DSS are characterised by plasma leakage which can result from severe dengue fever.

The haematocrit concentration (%) of children on the day of enrolment was recorded and saved in the hct_bsl column of the VDengue dataset. A daily haematocrit concentration count was evaluated between the third and eighth days of enrolment, and the maximum counts within those five days were noted and kept in the maxHCT_3to8 column of the dataset. On the same way, the overall haemoconcentration (%) was recorded in the maxhemo_3to8 column of the dataset. The data of patients who were admitted to the paediatric intensive care unit were stored in the to_PICU column (PICU).

**Figure 11:**
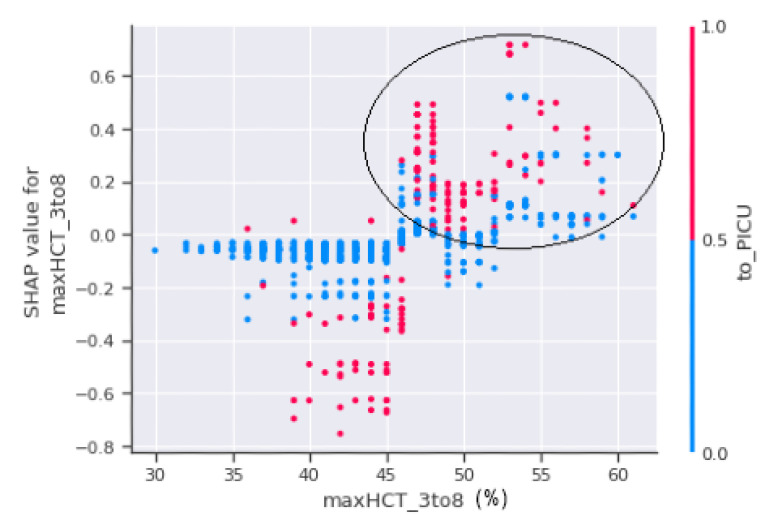
SHAP dependence plot between maxHCT_3to8 and to_PICU.

The horizontal axis, maxHCT_3to8, is depicted in Figure 11 and represents the actual value of the maximum haematocrit count between the third and eighth days of the patient’s enrolment, whereas the vertical axis represents a value that has an impact on the prediction, i.e., severity, to confirm shock or non-shock. Patients with a haematocrit concentration of more than 45% are more prone to fall into shock and develop DSS, as seen in the circled part of Figure 11. The circled region has a higher concentration of red dots which indicate that the patients in those areas were sent to the PICU.

**Figure 12:**
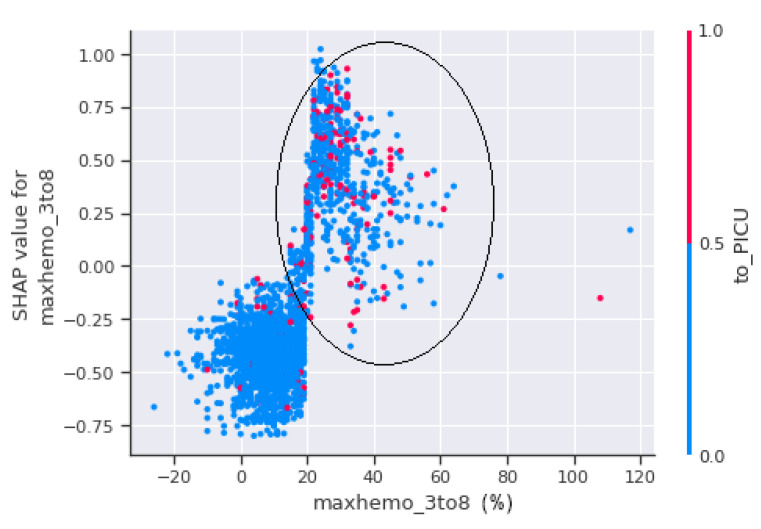
SHAP dependence plot between maxhemo_3to8 and to_PICU.

A scatter plot between maxhemo_3to8 and to_PICU is seen in Figure 12. Figure 12 shows that the patients with a haemoconcentration of more than 20% have the highest risk of suffering shock, regardless of whether they are admitted to PICU or not.

**Figure 13:**
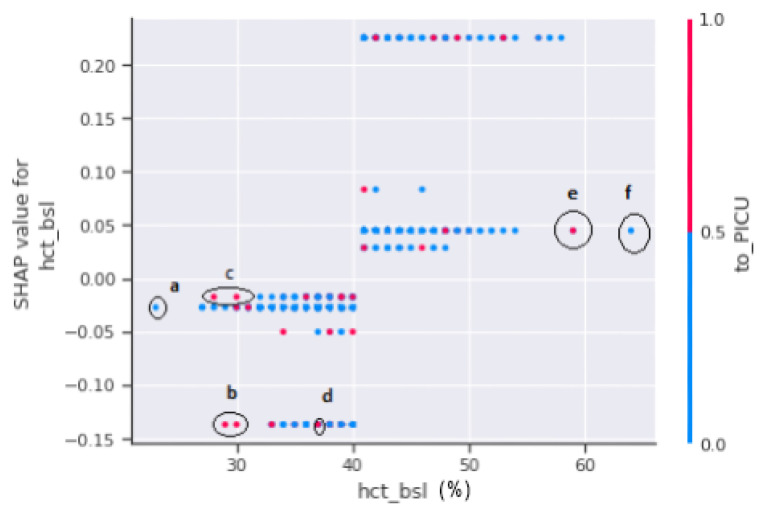
SHAP dependence plot between hct_bsl and to_PICU.

A SHAP dependency scatter plot between hct_bsl and to_PICU is shown in Figure 13. Haematocrit (HCT) testing was performed to determine the extent of plasma leakages. The SHAP-dependent plot in Figure 13 does not offer a clear picture. As a result, no decision can be made on the severity of the patients since, if marked regions “b”, “c”, and “d” are observed, any patient in the range of 25% to 45% of the hct concentration is either referred to PICU or has a minimal chance of progressing to shock. On the other hand, if marked regions “e” and “f” are considered, patients with hct levels exceeding 45% have a higher risk of developing dengue shock syndrome.

**Figure 14:**
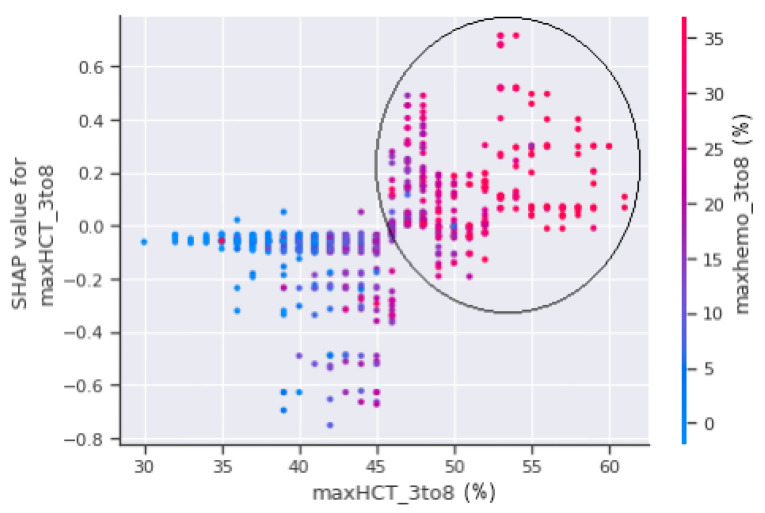
SHAP dependence plot between maxHCT_3to8 and maxhemo_3to8.

Figure 14 shows a relation between the maximum haematocrit count and the haemoconcentration on the third and eighth days of enrolment of the patients. The circle portion indicates, if the haemoconcentration is above 20% with respect to a 45% maximum haematocrit count, the patient is going to suffer from hazardous health issues, which may lead to either DHF or DSS.

**Figure 15:**
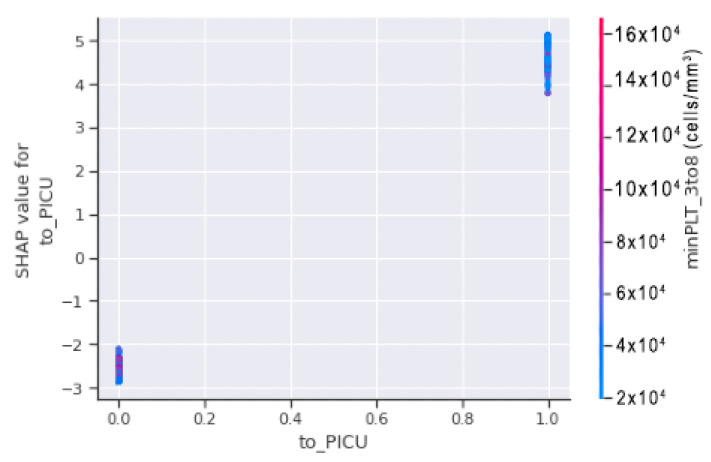
SHAP dependence plot of minPLT_3to8 and to_PICU illustrating to_PICU along horizontal axis and minPLT_3to8 along vertical axis.

- TO_PICU AND MINPLT_3TO8:

In the Figure 15, the horizontal axis contains to_PICU which shows the actual value from the datasets, and the vertical axis indicates the value of minPLT_3to8 that has an impact on the prediction. The increasing slope reflects the value of to_PICU, which is 1 (positive), indicating that the model is more likely to predict that the patient would go into shock.

**Figure 16:**
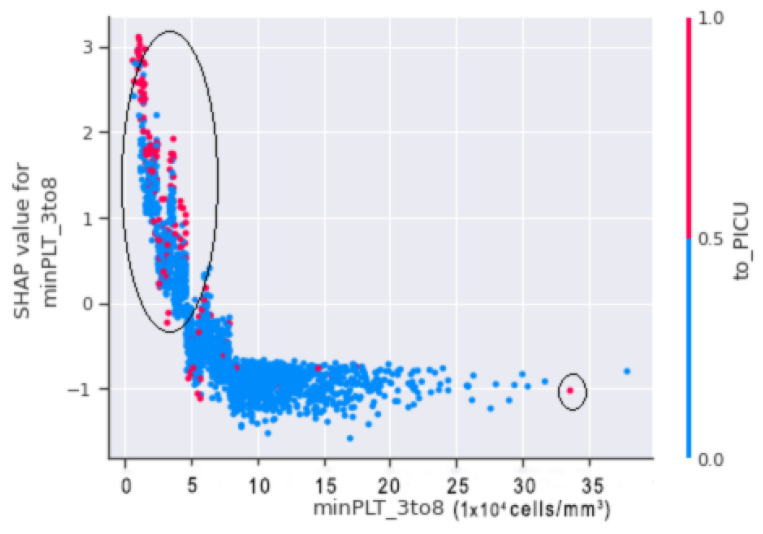
SHAP dependence plot of minPLT_3to8 and to_PICU illustrating minPLT_3to8 along horizontal axis and to_PICU along vertical axis.

In Figure 16, horizontal axis minPLT_3to8 refers to the actual value from the dataset, and the vertical axis shows the value that has an impact on the prediction. The upward slope shows that, when the value of to_PICU is 1 (positive), there is a higher chance for the patient to go into shock.

Thus, by merging both scatter plots of Figure 15 and Figure 16, it is shown that, when minPLT 3to8, i.e., patients with a minimal platelet count between the third and eighth days of admission to the hospital, have low values, there is a higher chance that the patient will go into shock. Furthermore, the majority of the children in the VDengue dataset who were on the verge of DSS or were in susceptible situations were admitted to the PICU. In the circled region of Figure 16, we can observe that the patients brought to the PICU had platelet counts ranging from 15,000 to 50,000 (cells/mm${}^{3}$), resulting in a positive SHAP score, indicating DSS. If we look at the right bottom corner, we can observe that the patient has taken to_PICU, however, since the platelet count is approximately 300,000 cells/mm${}^{3}$ and the patient has a negative SHAP value, it is unlikely that the patient would go into shock.

- MINPLT_3TO8 AND MAXHEMO_3TO8:

A SHAP dependence plot was plotted between minPLT_3to8 and maxhemo_3to8 to show the relation between the minimum platelets count and the maximum haemoconcentration from our datasets.

**Figure 17:**
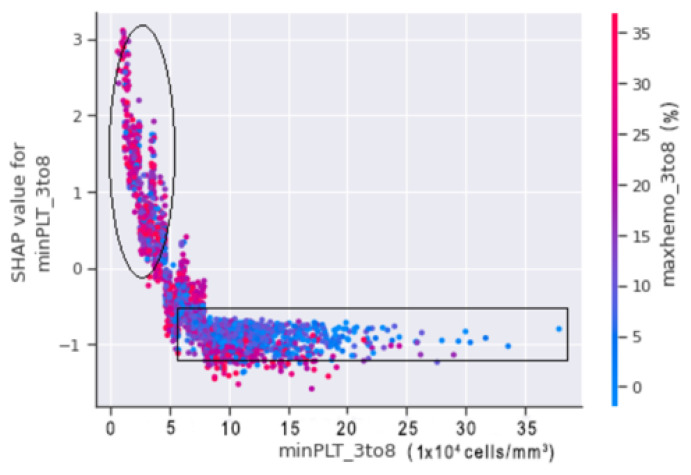
SHAP dependence plot between minPLT_3to8 and maxhemo_3to8.

In Figure 17, the circled region indicates that the patients in that region are more likely to fall into shock. Patients with a minimum platelet count ranges between 20,000 (cells/mm${}^{3}$) and 50,000 (cells/mm${}^{3}$) with a haemoconcentration of more than 20% are at risk of shock. A minimum platelet count of more than 50,000 (cells/mm${}^{3}$) and haemoconcentration of less than 20%, on the other hand, indicate that the patient is less prone to suffer DSS.

**Figure 18:**
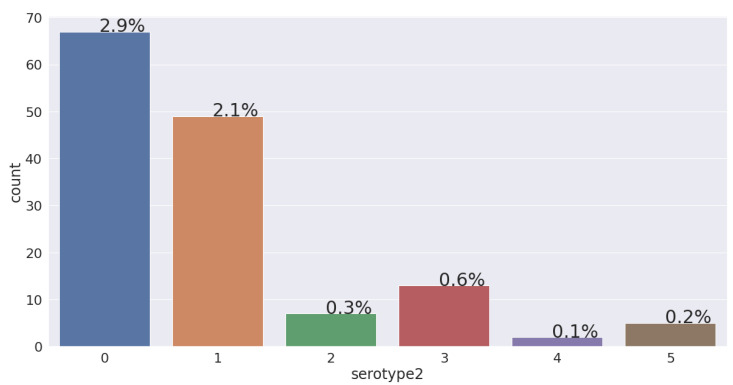
Percentage of serotype2 of patients going into shock.

- SEROTYPE2:

The serotype is the recognisable variation in bacteria or viruses or immune cells of different individuals within a species [28]. Considering the VDengue dataset, 6.21% of patients have reached to shock and have a tendency to develop dengue shock syndrome. If we look into Figure 18, we can see the percentage of the patients who have suffered from different serotypes. Among the 143 (6.21%) patients that reached to shock in the VDengue dataset, 67 (2.9%) had DENV1, 49 (2.1%) had DENV2, 7 (0.3%) had DENV3, 13 (0.6%) had DENV4, 2 (0.1%) had mixed serotypes, and 5 (0.2%) had no serotype. A bar diagram is also plotted based on the VDengue dataset, which shows that patients with DENV1 and DENV2 are most likely to associate with shock, where the patients have a high chance to develop DSS.

Taking into consideration the minimum platelets, the maximum haematocrit, and the haemoconcentration monitored for 3 to 8 days after admission into the hospital, the mean value for all the three features with respect to the serotype was calculated. After the calculation, it was found that the patients fell into the category of the DENV-1 serotype, and having a mean value of 30,942 (cells/mm${}^{3}$) platelets, 48% hematocrit, and 25% hemoconcentration is very much likely to lead to developing DSS.

In Figure 18,

- 0 = DENV1;
- 1 = DENV2;
- 2 = DENV3;
- 3 = DENV4;
- 4 = MIXED;
- 5 = NEGATIVE.

**Figure 19:**
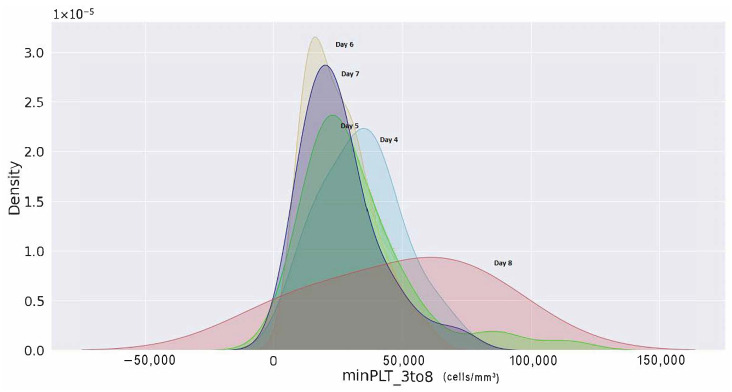
Minimum platelets count of shock victims as plotted each day since enrolment to the hospital using kernel density estimation plot.

**Figure 20:**
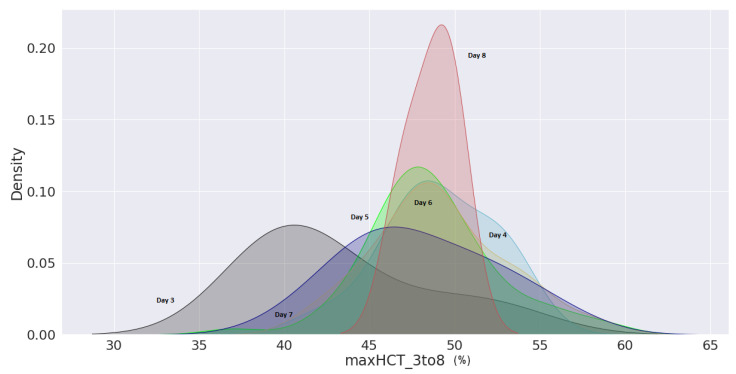
Maximum haematocrit concentration of shock victims as plotted each day since enrolment to the hospital using kernel density estimation plot.

Furthermore, a kernel density estimation graph was generated to assess the probability of minimum platelets and a maximum haematocrit count from day 3 to day 8 of their enrolment. The probability density of minimal platelets and highest haematocrit concentration of patients with shock are displayed in Figure 19 from day 3 of hospital admission to day 8. According to the density curve in Figure 19, patients with platelets fewer than 50,000 (cells/mm${}^{3}$) on day 6 counted from the day of patient registration had the highest density probability. Similarly, it can be shown in Figure 20 that an individual with haematocrit concentrations greater than 45% on day 8 had the highest density probability.

### 7.2. Analysis on the BDengue Dataset

The BDengue dataset was used with the agglomerative hierarchical clustering to determine different clusters of the patients. After fitting the dataset with the model, it starts to process by finding all of the dissimilarities between the data points. Two clusters could be formed after applying hierarchical clustering. The mean and standard deviation values of all features for both the clusters are shown in Table 6 and Table 7 after fitting the dataset with hierarchical clustering. Later, the two clusters were examined further to see whether or not patients from either cluster had progressed to severity [29]. Individual pair plots of two clusters were plotted to obtain insight into the patients displaying severity. The severity determination was made in accordance with WHO guidelines. Thus, after examining both clusters, 106 patients from a total of 169 patients belonged to cluster 0, and none of the patients from cluster 0 achieved severity since their platelet count was greater than 200,000 (cells/mm${}^{3}$).

Cluster 1 includes the remaining 63 cases. In the instance of Cluster 1, some individuals experience typical dengue fever whereas others are at risk of developing DHF. It was shown that 44% of cluster 1 patients have a very high likelihood of progressing to severity since their platelet count was less than 100,000 (cells/mm${}^{3}$), indicating that the patients may develop thrombocytopenia. In contrast, 55% of patients in cluster 1 have normal DF because their platelet count is greater than 100,000 (cells/mm${}^{3}$).

Moreover we can observe that the patients in cluster 0 have a mean neutrophils count of 65% and patients in cluster 1 have a mean neutrophils count of 52%. Neutrophils are the part of white blood cells that help the body to fight against any foreign body or any sort of infection and help the injured tissues to heal faster. However, individuals in cluster 1 are more likely to develop neutropenia, which is characterised by low levels of neutrophils, which increases the higher risk of getting infected by different types of infections [8]. In addition to that, the mean HCT percentage in cluster 1 patients is slightly higher than the cluster 0 patients. Despite having a higher risk of DHF or DSS, cluster 1 patients had a higher mean proportion of lymphocytes than cluster 0 patients. Cluster 0 has a mean percentage of lymphocytes count of 28.5%, whereas cluster 1 has a mean percentage of 40%. If the number of lymphocytes decreases further in cluster 0 individuals, lymphocytopenia may ensue. Furthermore, the mean percentage count of Hb and monocytes for both clusters was the same.

### 7.3. Correlation Study of BDengue and VDengue Datasets

We discovered a high link between DSS or DHF and the patient platelet count and HCT concentration in our study. According to our findings, individuals infected with dengue who had a platelet count of less than 100,000 (cells/mm${}^{3}$) have a greater risk of developing DSS or DHF, which we found in both the VDengue and BDengue datasets. Furthermore, based on the VDengue dataset, we observed that the HCT concentrations greater than 20% are associated with DSS or DHF.

## 8. Discussion

The main objective of our study was to apply different machine learning algorithms to predict severity among dengue-infected patients. We focused on the datasets (VDengue and BDengue) of two subtropical regions, where we applied several supervised learning methods to the VDengue dataset and, among them, the XGBoost classifier was found to be the best performing. Later, we implemented a SHAP dependence plot to see the effect of different features of the VDengue dataset on the prediction model, where we found that the platelets count and HCT concentration have greater effects on deciding whether the patient will proceed towards shock or not. On the other hand we applied unsupervised learning to the BDengue dataset, which included hierarchical clustering, from which, we deduced patients falling in the severity cluster had a lower platelets count and had an HCT concentration of more than 20%.

In the WHO, they have used a clinical approach to determine the factors that lead to Dengue Haemorrhagic Fever (DHF) and Dengue Shock Syndrome (DSS). According to the 1997 WHO case definition, patients with thrombocytopenia (≤100,000 cells/mm${}^{3}$) and evidence of plasma leakage (at least a rise in haematocrit of ≥20% compared with the baseline value of the patient) or other signs of plasma leakage (such as pleural effusion and/or ascites) are classified to have DHF, and, on the other hand, all four symptoms including shock being present in DSS [8].

We reached a similar conclusion to the WHO guidelines from both the VDengue and BDengue datasets using a machine learning approach following two different processes, one for a structured dataset and another for an unstructured dataset. Our findings support the findings by WHO.

In the future, we intend to overcome the limitations faced, such as unstructured datasets, unwillingness to share patient data, paper-based records, and missing attributes, that would help to determine time series. If we can overcome the aforementioned limitations, it will be easier for us to identify the DSS or DHF patients more accurately. Thus, healthcare professionals will be able to take proper countermeasures and make necessary arrangements for the dengue-infected patients.

## Figures and Tables

**Table 1:** Attributes of VDengue dataset.

Name of the Attributes	Description
st_no	Patient study number
age	Age at enrolment (years)
sex	Gender (Female, Male)
wt	Weight at enrolment (kg)
day_ill	Day of illness at enrolment
his_tired	History of tiredness at enrolment (Yes, No)
his_vomit	History of vomit at enrolment (Yes, No)
ttest	Tourniquet test result at enrolment (Positive, Equivocal, Negative)
temp	Temperature at enrolment (${}^{\circ }$C)
pulse	Pulse rate at enrolment (count per minute)
sys_bp	Systolic blood pressure at enrolment (mmHg)
mucosal_bleed	Mucosal bleeding at enrolment (Yes, No)
abdominal_pain	Abdominal pain at enrolment (Yes, No)
liver	Liver sice at enrolment (cm)
hct_bsl	Haematocrit concentration at enrolment (%)
plt_bsl	Platelets count at enrolment (cells/mm${}^{3}$)
serotype2	Serotype determined by PCR (DENV1, DENV2, DENV3,Mixed, Negative)
serology	Immune status determined by ELISA (Primary, Secondary, Possible Primary, Unclassifiable)
to_PICU	Referred to PICU (Yes, No)
shock	Dengue shock syndrome (Yes, No)
doi_shock	Day of illness at shock (days)
bleed_hos	Bleeding during hospitalisation (No, Skin, Mucose, Other)
minPLT_3to8	Platelet nadir (cells/mm${}^{3}$) (No, Skin, Mucose, Other)

**Table 2:** Attributes of dataset collected from Dr. M. R. khan Shishu Hospital (Bangladesh).

Name of the Attributes	Description	Unit
DOA	Date of admission of patient	-
PatientId	ID of patients enrolled in the hospital	-
PatientName	Name of the patient	-
Gender	Gender (Female or Male)	-
Age	Age of the patient	years
Hb	Haemoglobin count	g/dL
TotalCountWBC	Total WBC count of the patient	-
Platelets	Platelets count of patient	cells/mm${}^{3}$
ESR	Erythrocyte sedimentation rate of patient	mm
DengueNS1	Dengue virus antigen detection	-
WeightOfThePatient	Weight of the patient	kg
BloodPressure	Blood pressure of patient	mm/Hg
HCT	Haematocrit concentration of patient	%
Lymphocytes	-	%
Monocytes	-	%
Neutrophils	-	%
Eosinophils	-	%
Basophils	-	%
BloodGroup	Blood group of the patient	-
Dengue IgM/IgG	Antibody testing in dengue diagnosis	-
SGPT	SGPT of patient	-
Albumin	-	m/dL
Symptoms	Symptoms of the patient	-

**Table 3:** Attributes of dataset collected from central hospital (Bangladesh).

Name of the Attributes	Unit	Name of the Attributes	Unit
Date Of Arrival	dd/mm/yy	Monocytes	%
Patient ID	-	Basophils	%
Patient Name	-	HCT	%
Gender	0(M)/1(F)	MCV	fl
Age	year	MCH	pg
Haemoglobin	g/dL	MCHC	g/dL
WBC Count	/cmm	RBC Count	million/cm
Platelets	K/L	Dengue NS1	Positive/Negative
Neutrophils	%	Dengue IgG	Positive/Negative
Lymphocytes	%	Dengue IgM	Positive/Negative
Eosinophils	%	-	-

**Table 4:** Classification report after applying different machine learning approaches on the VDengue dataset.

Algorithm	Misclassification	Precision	f1_Score	PPV	NPV
AdaBoost	0.02	0.98	0.96	0.74	1
XGBoost	0.01	0.98	0.96	0.8	1
Random Forest	0.02	0.98	0.96	0.73	1
Decision Tree	0.02	0.98	0.96	0.76	1

**Table 5:** Training accuracy, testing accuracy, sensitivity, and specificity of different models on VDengue dataset.

Algorithm	Training Accuracy	Test Accuracy	Sensitivity	Specificity
AdaBoost	0.998	0.981	0.94	0.98
XGBoost	1	0.986	0.94	0.99
Random Forest	1	0.979	0.94	0.98
Decision Tree	1	0.982	0.94	0.98

**Table 6:** Mean and standard deviation of different features for cluster 0.

Attributes	Mean	Standard Deviation
Platelets (cells/mm${}^{3}$)	221,085	63,918
HCT (%)	37.94	4.77
Lymphocytes (%)	28.50	16.26
Monocytes (%)	4	1.99
Neutrophils (%)	65	18.16
WBC	6918	5812
Hb (g/dL)	12	1.61

**Table 7:** Mean and standard deviation of different features for cluster 1.

Attributes	Mean	Standard Deviation
Platelets (cells/mm${}^{3}$)	93,714	3596
HCT (%)	40.63	5.96
Lymphocytes (%)	40	18.57
Monocytes (%)	4	1.95
Neutrophils (%)	52	19.82
WBC	10,521	18,762
Hb (g/dL)	12	2.02

## Data Availability

We have used two datasets in this research study. The VDengue dataset is publicly available at [5]. The BDengue dataset was collected from two different hospitals and will be made available on request.

## Abbreviations

The following abbreviations are used in this manuscript:

DHF	Dengue Haemorrhagic Fever
DSS	Dengue Shock Syndrome
HCT	Haematocrit
KNN	K-Nearest Neighbours
MICE	Multivariate Imputation By Chained Equations
PICU	Paediatric Intensive Care Unit
SHAP	SHapley Additive exPlanations
WBC	White Blood Cell
XG	eXtreme Gradient
